# The blink reflex magnitude is continuously adjusted according to both current and predicted stimulus position with respect to the face

**DOI:** 10.1016/j.cortex.2016.04.009

**Published:** 2016-08

**Authors:** Sarah B. Wallwork, Kerwin Talbot, Danny Camfferman, G.L. Moseley, G.D. Iannetti

**Affiliations:** a Sansom Institute for Health Research, Division of Health Sciences, University of South Australia, Adelaide, Australia; b Department of Neuroscience, Physiology and Pharmacology, University College London, London, United Kingdom; c Neuroscience Research Australia, Sydney, Australia

**Keywords:** Nervous system, Blink reflex, Threat detection, Brainstem, Top-down modulation

## Abstract

The magnitude of the hand-blink reflex (HBR), a subcortical defensive reflex elicited by the electrical stimulation of the median nerve, is increased when the stimulated hand is close to the face (‘far–near effect’). This enhancement occurs through a cortico-bulbar facilitation of the polysynaptic medullary pathways subserving the reflex. Here, in two experiments, we investigated the temporal characteristics of this facilitation, and its adjustment during voluntary movement of the stimulated hand. Given that individuals navigate in a fast changing environment, one would expect the cortico-bulbar modulation of this response to adjust rapidly, and as a function of the predicted spatial position of external threats. We observed two main results. First, the HBR modulation occurs without a temporal delay between when the hand has reached the stimulation position and when the stimulus happens (Experiments 1 and 2). Second, the voluntary movement of the hand interacts with the ‘far–near effect’: stimuli delivered when the hand is far from the face elicit an enhanced HBR if the hand is being moved towards the face, whereas stimuli delivered when the hand is near the face elicit an enhanced HBR regardless of the direction of the hand movement (Experiment 2). These results indicate that the top-down modulation of this subcortical defensive reflex occurs continuously, and takes into account both the current and the predicted position of potential threats with respect to the body. The continuous control of the excitability of subcortical reflex circuits ensures appropriate adjustment of defensive responses in a rapidly-changing sensory environment.

## 1 Introduction

The eye blink elicited by electrical stimulation of the median nerve at the wrist hand-blink reflex (HBR) is a defensive reflex subserved by an entirely subcortical circuit at brainstem level (Miwa et al., 1998, Valls-Solé et al., 1997). Human electromyographic (EMG) recordings from the *orbicularis oculi* muscles show that the HBR consists of a bilateral response with an onset latency of ∼45 msec. The HBR is functionally similar to the R2 component of the trigemino-facial blink reflex (Cruccu & Deuschl, 2000).

The magnitude of the HBR increases when the proximity between the stimulated hand and the face is reduced (Sambo et al., 2012b, Sambo et al., 2012a). Such increase has allowed the identification of a portion of space surrounding the face with a protective function, the defensive peripersonal space (DPPS) (Bufacchi et al., 2015, Sambo and Iannetti, 2013, Sambo et al., 2012a). Similarly to what has been observed in non-human primates (Graziano & Cooke, 2006), potentially harmful stimuli occurring within this space elicit stronger defensive responses compared to stimuli located outside of it (Sambo and Iannetti, 2013, de Vignemont and Iannetti, 2015).

The HBR enhancement is consequent to a tonic, cortico-bulbar facilitation of the polysynaptic medullary pathways that relay the somatosensory input to the facial nuclei at pontine level (Sambo, Forster, et al., 2012). The strength of this facilitation is determined by a number of cognitive factors, which demonstrates its defensive value; for example, the HBR magnitude increase is finely adjusted depending on the estimated probability that the threatening stimulus will occur, as well as on the presence of defensive objects near the face (Sambo, Forster, et al., 2012). These observations highlight the behavioural relevance of such fine top-down modulation of this subcortical reflex.

In contrast, the temporal dynamic of this top-down modulation has not been explored. Indeed, in previous experiments the eliciting stimuli were delivered using a long temporal interval (i.e., more than 20 sec) after the hand was placed at the target distance from the face (Sambo et al., 2012b, Sambo and Iannetti, 2013, Sambo et al., 2012a). Therefore, the only information about the temporal profile of the cortico-bulbar facilitation underlying the HBR increase is that it is exerted *tonically*, well before the eliciting stimulus is delivered (Sambo, Liang, et al., 2012).

Given that individuals navigate in a fast changing environment, one would expect the cortico-bulbar facilitation to adjust within a time frame appropriate to minimise the potential for harm of sudden external events (i.e., within tens of milliseconds), and as a function of the predicted spatial position of external threats. Here, in two experiments we investigated the temporal characteristics of the cortico-bulbar facilitatory effect (*Experiment 1*), and its adjustment depending on the predicted position of the stimulus (*Experiment 2*). In *Experiment 1* we exploited the well-established HBR enhancement observed when the stimulated hand is located inside the DPPS of the face (position ‘Near’) compared to when it is located outside (position ‘Far’). We tested whether the HBR enhancement is modulated by the length of the time interval between when the hand reached the target position and the subsequent delivery of the eliciting stimulus. In *Experiment 2* we exploited the ability of the nervous system to accurately predict limb positions during voluntary movement: participants continuously moved their hand between the ‘Far’ and ‘Near’ positions and the stimulus was automatically delivered either inside or outside the DPPS, when the hand was moving either towards or away from the face. We therefore tested whether the HBR facilitation depends on the *direction* of the movement of the stimulus with respect to the body.

## 2 Methods

### 2.1 Participants

Sixty six healthy participants were screened for this study, to identify HBR responders (Miwa et al., 1998). All participants gave written, informed consent before taking part in the study. All procedures were approved by the local ethics committee.

### 2.2 Stimulation and recording

Electrical stimuli were delivered to the right median nerve at the wrist using a bipolar surface electrode (inter-electrode distance: ∼2 cm) attached to a Digitimer constant current stimulator (model DS7A). Stimulus duration was 200 μsec. Stimulus intensity was adjusted, in each participant, to elicit a clear HBR in at least three consecutive trials (3.5–70 mA, mean ± SD: 16.7 ± 16.3 mA). The definition of a clear HBR was subjective, and based on the visual inspection of the EMG recording, as in previous HBR experiments (Sambo et al., 2012a, Valls-Solé et al., 1997). EMG activity was recorded from the orbicularis oculi muscle, bilaterally, using pairs of surface electrodes. The active electrode was located ∼1 cm below the lower eyelid, and the reference electrode ∼1 cm laterally of the outer canthus. Signals were amplified and digitized at a sampling rate of 10 kHz (Neuroscan 4.5). In *Experiment* 2, the position of the hand was continuously monitored using a 3D localizer (Polhemus Fastrak) programmed to trigger a stimulus when the hand reached two pre-defined positions, one inside and one outside the DPPS (see next section for details). This device allows localizing the position and orientation of the hand, and consists of an alternating current static magnetic transmitter that emits an electromagnetic dipole field. Tracking sensors were attached to the moving hand and to the forehead, and their positions were located relative to the position of the static transmitter.

### 2.3 Experimental procedures

#### 2.3.1 Preliminary recordings

Participants sat in a comfortable chair with their forearms resting on a pillow laying on a table in front of them. In each participant we first determined whether they were ‘responders’, by increasing the stimulus intensity until a clear HBR was elicited in three consecutive trials, or the participant refused a further increase of stimulus intensity (Valls-Solé et al., 1997). Participants with a reproducible HBR (i.e., responders, *N* = 37; 23 women, 18–63 years, mean ± SD: 25.3 ± 9.3 years) underwent further testing. The percentage of recruited subjects who were HBR responders (56%) was consistent with previous studies (Sambo et al., 2012b, Sambo et al., 2012a). During the experiments, participants were asked to keep their gaze fixed on a cross (4 × 4 cm) placed centrally in front of them, at a distance of ∼100 cm, 20 cm below eye level. White noise was played to mask any possible auditory cue about the incoming stimulation.

#### 2.3.2 Experiment 1

**Fig. 1:**
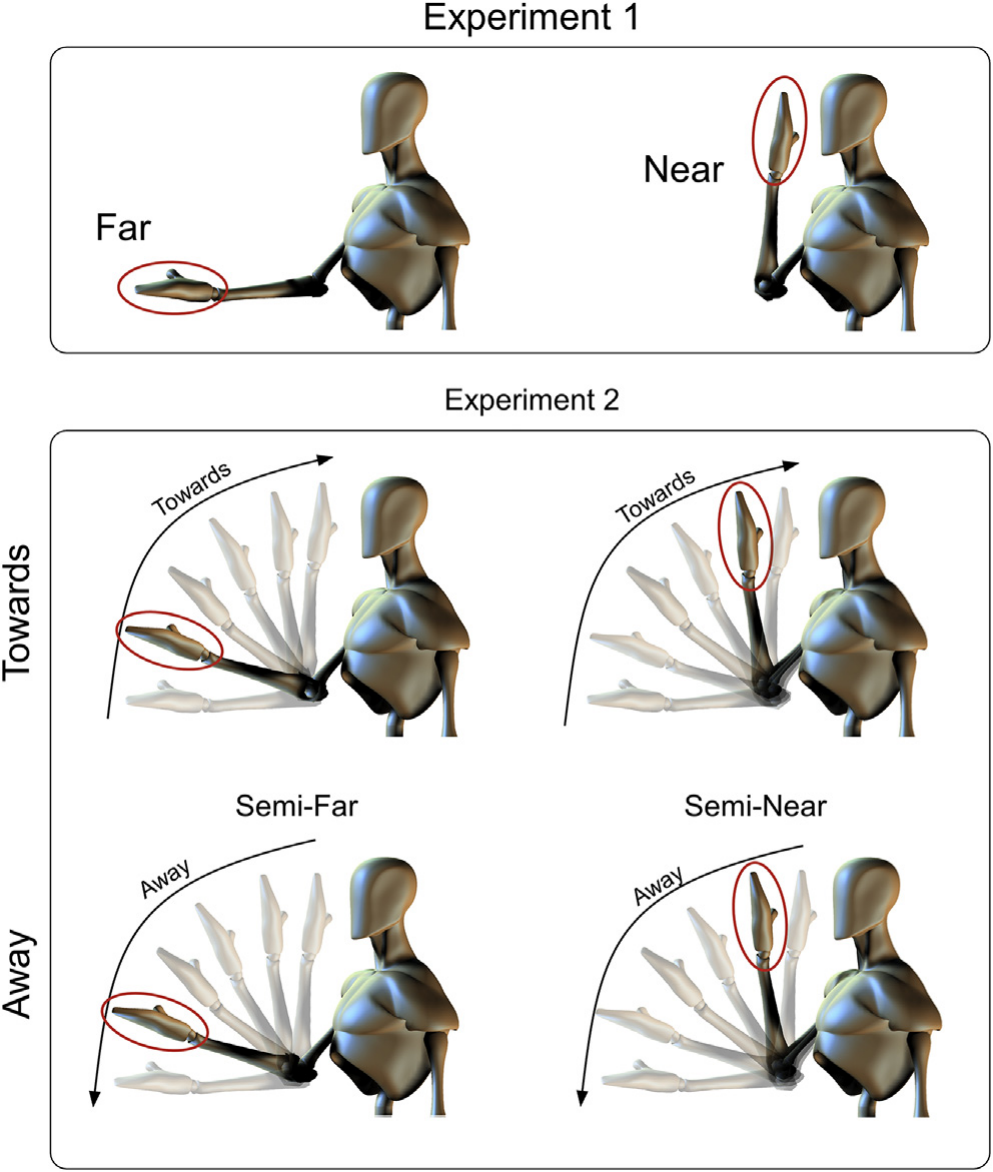
In Experiment 1 (top panel) the HBR was elicited by electrical stimulation of the median nerve at the wrist and recorded from the *orbicularis oculi* muscles, when the hand was in ‘Far’ and ‘Near’ positions with respect to the ipsilateral side of the face (see main text for details). In Experiment 2, the HBR was elicited when the hand was in ‘Semi-far’ and ‘Semi-near’ hand positions (see main text for details), while the hand was moving either towards or away from the face. Red ellipses indicate the location of the hand position when the stimulus was delivered.

In 17 responders we tested whether the ‘Far’–‘Near’ HBR enhancement was modulated by the length of the time interval between when the hand reached the target position and the subsequent delivery of the eliciting stimulus. Stimuli were delivered with the hand in two positions: either while the forearm was at ∼130° with respect to the arm, a posture resulting in the wrist being at a distance of ∼40–60 cm from the ipsilateral side of the face (position ‘Far’), or while the forearm was at ∼75° with respect to the arm, and the wrist at ∼4 cm from the ipsilateral side of the face (position ‘Near’). Stimuli were delivered with a delay of 2, 5, 10, or 30 sec after the hand reached the target position (‘Far’ or ‘Near’) (Fig. 1, upper panel). A total of 80 stimuli were delivered, in two blocks. In each block 5 stimuli were delivered for each position and delay, for a total of 40 stimuli. Stimuli were delivered in the ‘Far’ and ‘Near’ positions in alternating trials. The order of delays was pseudorandomised, with no more than two consecutive stimuli delivered at the same delay. At the beginning of each trial, participants were verbally instructed to place their hand in either the ‘Far’ or the ‘Near’ position, but they were not informed of the delay between when they placed the hand in the target position and stimulus delivery. The interval between two consecutive stimuli was ∼30 sec.

#### 2.3.3 Experiment 2

In 20 responders we tested whether the cortico-bulbar modulation of the HBR excitability depends on the direction of the movement of the stimulus with respect to the body. Stimuli were delivered with the hand in two positions: either while the forearm was at ∼100° with respect to the arm, a posture resulting in the wrist being at a distance of ∼40 cm from the ipsilateral side of the face (position ‘Semi-far’), or while the forearm was at ∼85° with respect to the arm, and the wrist at a distance of ∼13 cm from the ipsilateral side of the face (position ‘Semi-near’ – note that this position was different from position ‘Near’ of Experiment 1) (Fig. 1, lower panel).

Participants were instructed to move their hand between the positions ‘Far’ and ‘Near’ (i.e., the same positions of Experiment 1). Therefore, the trajectory between the ‘Far’ and ‘Near’ positions included the ‘Semi-far’ and ‘Semi-near’ at which the hand was stimulated (Fig. 1, lower panel). Participants were instructed to move the hand at constant speed, and the frequency of oscillation between the ‘Far’ and ‘Near’ positions was approximately .25 Hz (i.e., 2 sec to displace the hand from ‘Far’ to ‘Near’, and vice-versa). The position of the hand was continuously sampled using the 3D localizer, which triggered the electrical stimulus when the hand was in one of the two target positions. Participants received 10 stimuli at each stimulation position (‘Semi-far’ and ‘Semi-near’) and movement direction (‘Towards’ and ‘Away’), for a total of 40 stimuli. Stimuli delivered at ‘Semi-far’ and ‘Semi-near’ positions were alternated. Stimuli delivered while the hand was moving ‘Towards’ and ‘Away’ from the face were delivered in pseudorandom order, with no more than two consecutive stimuli delivered while the hand was moving in the same direction. The interval between two consecutive stimuli was always ∼30 sec.

### 2.4 Data analysis and statistics

EMG data were analysed using Neuroscan 4.5, MATLAB and Letswave 5 (www.nocions.org/letswave) (Mouraux & Iannetti, 2008). EMG signals from each participant were high-pass filtered (55 Hz), full wave rectified, and averaged across ipsilateral and contralateral recording sides. HBR responses were averaged separately for each subject and experimental condition. Statistical analyses were conducted on low-pass filtered (200 Hz) waveforms, at each time point of the averaged EMG waveform, for each participant.

In *Experiment 1*, we performed a two-way, repeated-measures ANOVA, with ‘Position’ (two levels: Far and Near) and ‘Time’ (four levels: 2, 5, 10, and 30 sec) as experimental factors. In *Experiment 2*, we performed a two-way, repeated-measures ANOVA with ‘Position’ (two levels: Far and Near) and ‘Movement’ (two levels: Towards and Away) as experimental factors. To investigate the time course of the possible effects of these experimental factors, the ANOVA was performed on each time point of the averaged HBR (as implemented in Letswave) (Mouraux & Iannetti, 2008). Such a point-by-point ANOVA yielded a waveform expressing the significance of the effect of each factor, as well as of their interactions across the time course of the HBR response. When main effects or interactions were significant, Bonferroni-corrected *post hoc* paired t-tests were performed. A consecutivity threshold of 10 msec was chosen to account for multiple comparisons, as in Sambo, Forster, et al. (2012) and in Sambo and Iannetti (2013). Statistical significance was set at .05.

## 3 Results

### 3.1 Experiment 1

**Fig. 2:**
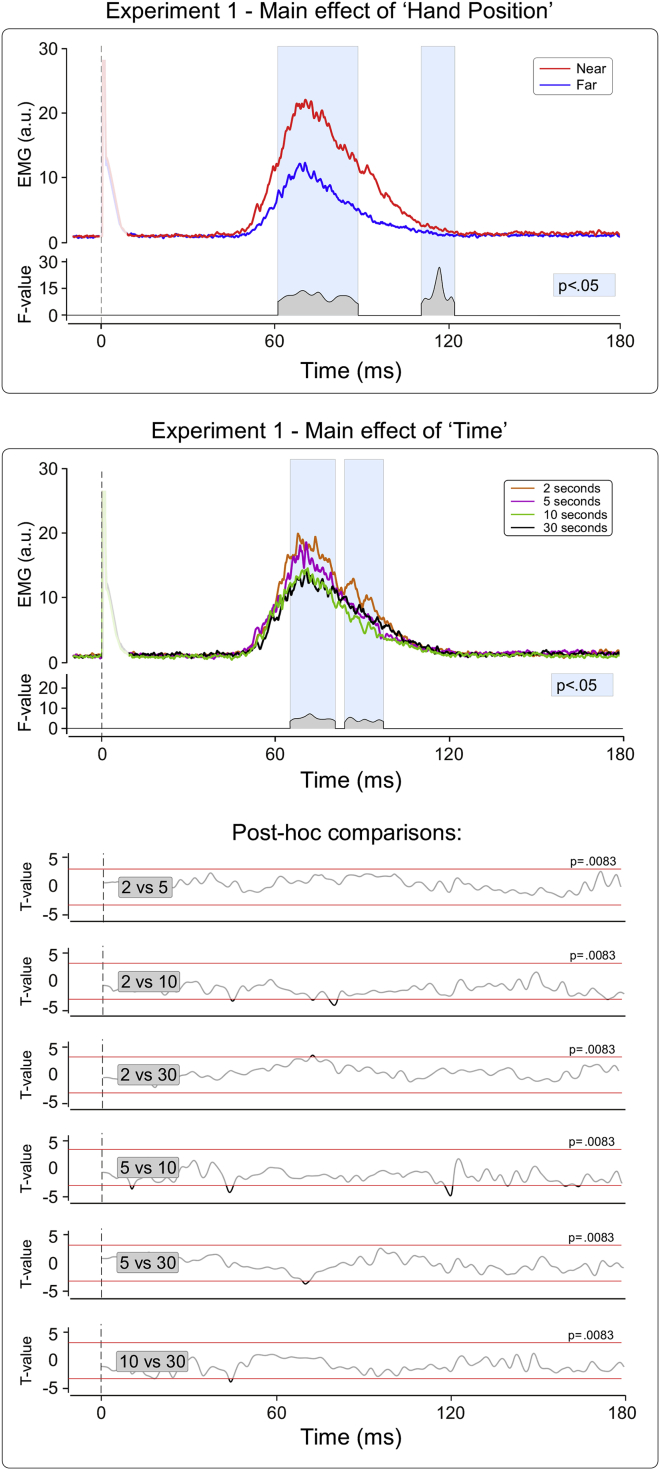
Experiment 1. Main effects of the experimental factors ‘Hand Position’ (two levels: Far, Near) and ‘Time’ (four levels: 2, 5, 10, 30). In each panel, the top waveforms are the rectified, group-average HBR for the levels of each factor; the bottom waveforms express the *F*-value of the two-way ANOVA for each time point, in the significant time windows (*p* < .05). The *t*-value waveforms show the six post hoc comparisons exploring the effect of ‘Time’ at different delays. The red lines denotes the threshold for significance, corrected for multiple comparisons (*p* = .0083). These results show that the top-down cortical modulation underlying the HBR enhancement is similar at the four explored delays.

In Experiment 1 we tested whether the HBR enhancement due to the stimulated hand being located inside the DPPS of the face (factor ‘Position’) was modulated by how long the hand was kept in the target position before receiving the successive stimulus (factor ‘Time’). The factor ‘Position’ was a significant source of variance within two time windows: 60–89 and 111–123 msec post-stimulus (*p* < .05; see Fig. 2 for the *F*-value timecourse). This indicates that the HBR magnitude was overall larger when the stimulated hand was inside the DPPS of the face than when it was outside, thus confirming a number of previous observations (Sambo et al., 2012b, Sambo and Iannetti, 2013, Sambo et al., 2012a). The factor ‘Time’ was a significant source of variance within two time windows: 65–81 and 84–98 msec post-stimulus (*p* < .05, see Fig. 2 for the *F-*value timecourse). Post hoc paired t-tests between the four levels of the factor ‘Time’ revealed no significant differences between all pairs of time delays (Fig. 2). Crucially, there was no ‘Position’ × ‘Time’ interaction (*p* > .05 for all time points), indicating that the HBR increase in the ‘Near’ position was similar at the four explored time delays.

The results of Experiment 1 indicate that the top-down cortical modulation underlying the HBR enhancement is similar at the four explored delays, and therefore can occur as quickly as 2 sec from when the hand is placed in the stimulated position.

### 3.2 Experiment 2

**Fig. 3:**
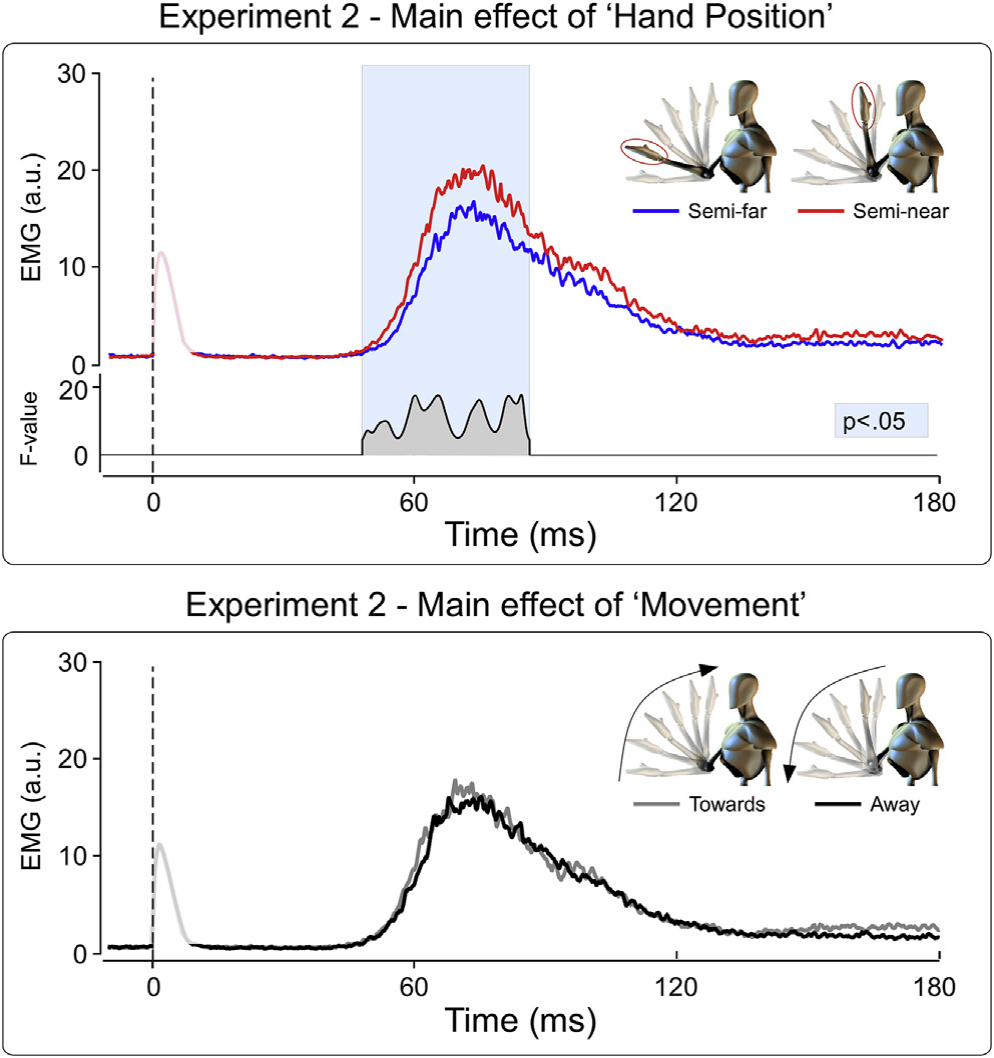
Experiment 2. Main effects of the experimental factors ‘Hand Position’ (two levels: Semi-far, Semi-near) and ‘Movement’ (two levels: Towards, Away). In the upper panel, the top waveforms are the rectified, group-average HBR for the Semi-far and Semi-near levels; the bottom waveform expresses the F-value of the two-way ANOVA for each time point, in the significant time windows (*p* < .05). In the lower panel the waveforms are the group-average HBR for the Towards and Away levels of the factor ‘Movement’.

**Fig. 4:**
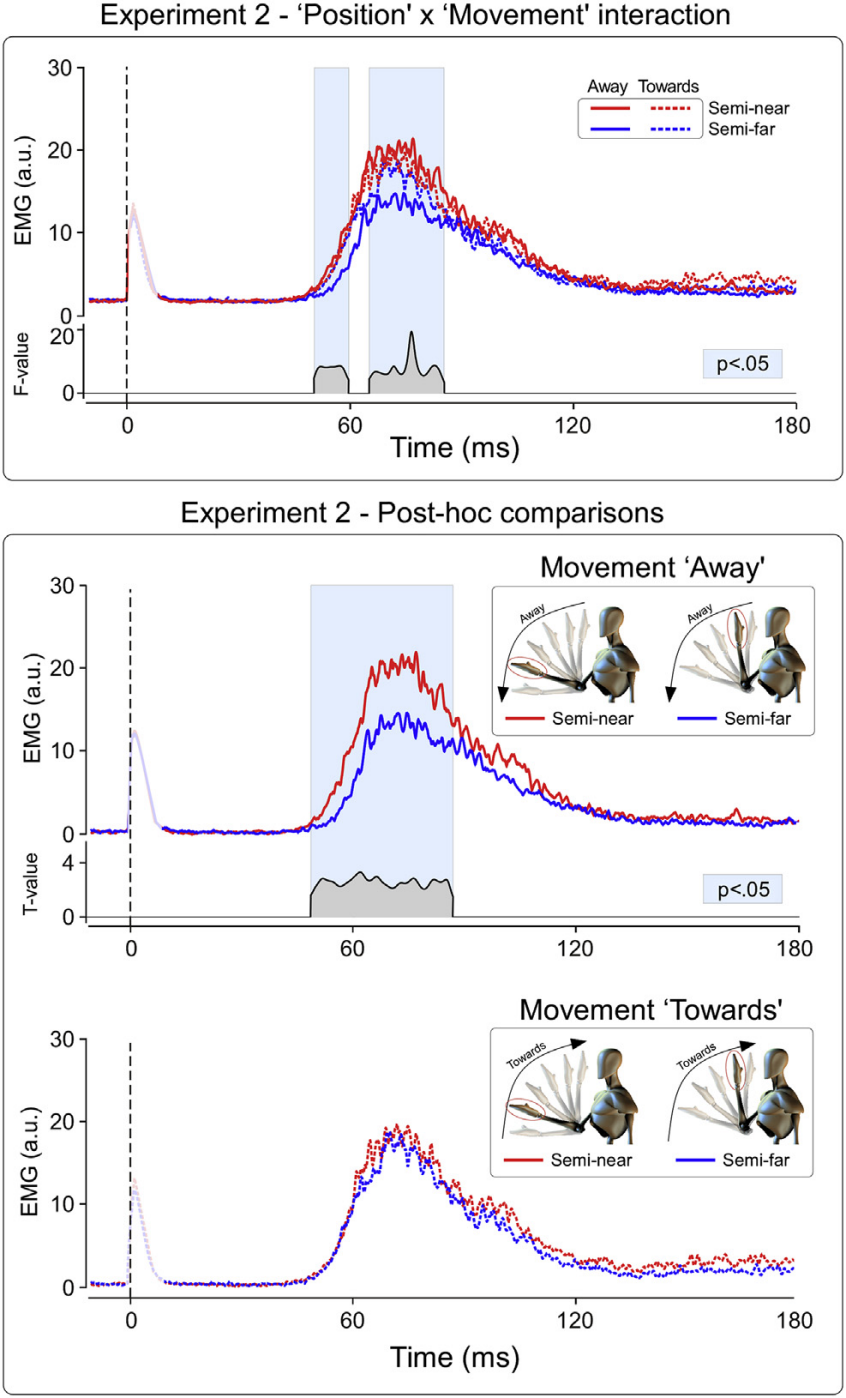
Experiment 2. ‘Position’ × ‘Movement’ interaction. Upper panel: the top waveforms are the rectified, group-average HBR in the four experimental conditions; the bottom waveform expresses the F-value of the interaction term, in the significant time windows (*p* < .05). Lower panel: post-hoc paired t-tests, comparing the HBR responses elicited while the hand was in the ‘Semi-near’ and ‘Semi-far’ locations, for both ‘Away’ (top) and ‘Towards’ (bottom) directions. Only in the ‘Away’ condition the HBR was significantly larger when the hand was in position ‘Semi-near’ than in position ‘Semi-far’. This interaction indicates that the excitability of the medullary circuit mediating the HBR depends on both the current and the predicted direction of the movement of the threat in respect to the body. When the hand is moving towards the face, the stimulus threat value is increased, resulting in a large HBR even if the actual hand position is ‘Semi-far’.

In Experiment 2 we tested whether the cortico-bulbar modulation of the HBR excitability depends on the predicted position of the stimulus, as well as by the direction of stimulus movement (towards or away from the body). The factor ‘Position’ was a significant source of variance within the 49–87 msec post-stimulus time window (*p* < .05; Fig. 3, upper panel), while the factor ‘Movement’ was not (Fig. 3, lower panel). Crucially, there was a significant ‘Position’ × ‘Movement’ interaction within two time windows: 51–61 and 66–86 msec post-stimulus (*p* < .05; Fig. 4, upper panel). We explored this interaction by performing two post-hoc paired t-tests, comparing the HBR responses elicited while the hand was in the ‘Semi-near’ and ‘Semi-far’ positions, for both ‘Towards’ and ‘Away’ movement directions. In the ‘Away’ condition, HBR was significantly greater when the hand was in position ‘Semi-near’ than in position ‘Semi-far’ (48–88 msec post-stimulus; Fig. 4, lower panel), thus reproducing the previously observed increase of HBR magnitude while the hand is close to the face (Sambo et al., 2012b, Sambo and Iannetti, 2013). In contrast, in the ‘Towards’ condition, the HBR was not different in the ‘Semi-far’ and ‘Semi-near’ positions, because of a larger HBR in the ‘Semi-far’ position (Fig. 4, lower panel). This finding indicates that (1) the excitability of the medullary circuit mediating the HBR is continuously adjusted as a function of the predicted hand position, and (2) this prediction depends on the direction of the movement of the threat with respect to the body. When the hand is moving towards the face, the threat value is increased, resulting in a large HBR even if the actual hand position is ‘Semi-far’.

## 4 Discussion

In this study we investigated the temporal characteristics of the cortico-bulbar modulation of the brainstem circuits mediating the HBR, as well as their dependency on the predicted position of the stimulated hand during a voluntary movement.

We observed three main findings. First, the top-down cortical modulation of the medullary circuitry subserving the HBR occurs as quickly as 2 sec from when the hand is placed in the stimulated position (*Experiment 1*). Second, it is continuously adjusted as a function of both the current and predicted hand position (*Experiment 2*). Third, it depends on the *direction* of the movement of the stimulus with respect to the body (*Experiment 2*): the hand movement towards the face results in a large HBR even if the actual hand position is far from the face. This is consistent with the notion that a stimulus approaching the body has a higher threat value.

These findings indicate that the central nervous system is able to rapidly adjust the excitability of subcortical defensive responses, and thereby exploit the predictions about the spatial location of the threatening stimulus in a purposeful manner. These modulations take into account both the current and predicted position of a potential threat in respect to the body. This neural mechanism ensures appropriate adjustment of defensive responses in a rapidly-changing sensory environment.

### 4.1 Top-down HBR modulation occurs rapidly

Experiment 1 showed that the HBR enhancement observed when the stimulated hand is located near the face (Sambo et al., 2012b, Sambo et al., 2012a) occurs within two seconds from when the hand has been in position prior to receiving the stimulus. Indeed, there were no differences in the ‘Far’–‘Near’ effect across the four time delays explored (Fig. 2). Experiment 2 further characterised the temporal properties of the HBR enhancement: the HBR magnitude was modulated continuously as a function of both the current and the predicted position of the stimulated hand with respect to the face. This, together with the previous evidence that the brainstem medullary interneurons subserving the HBR response are under cortico-bulbar control (see Fig. 2 in Sambo, Forster, et al., 2012), indicates that such top-down modulation is continuously and purposefully regulated (Fig. 4).

It is well-known that the blink reflex can be cognitively modulated at short time scales. For example, Codispoti, Bradley, and Lang (2001) observed that the blink reflex elicited by an auditory stimulus is enhanced by the presentation of an unpleasant image preceding the auditory stimulus by as short as 300 msec. Similarly, Ehrlichman, Brown, Zhu, and Warrenburg (1995) showed that the blink reflex is increased when the eliciting auditory stimulus is preceded by an unpleasant odour by 400 msec. However, these modulations entailed emotional stimuli which are known to alter the arousal level and generally facilitate motor responses (Lang, Greenwald, Bradley, & Hamm, 1993). In contrast, the cortico-bulbar modulation underlying the HBR enhancement reported in the current study is specific for the medullary interneurons receiving somatosensory input from the stimulated hand (i.e., it is not consequent to a facilitation of motor output from the nucleus of the VII cranial nerve, or to a general increase of excitability of the medullary interneurons mediating the blink reflex elicited by other somatosensory stimuli; Sambo, Forster, et al., 2012). Therefore, on the basis of the proprioceptive and visual information about the spatial location of the stimulated hand, the nervous system remaps the respective position of the hand and the face onto the same external reference frame, and thereby infers their distance. This distance estimate is used to adjust the cortical modulation of medullary circuits subserving the HBR.

Therefore, on the basis of the proprioceptive and visual information about the spatial location of the stimulated hand, the nervous system remaps the respective position of the hand and the face onto the same reference frame, and thereby infers their distance. This distance estimate is used to adjust the cortical modulation of medullary circuits subserving the HBR.

The fact that the excitability of defensive reflexes is continuously adjusted depending on the position of the threats with respect to the body has a clear survival value, as such reflexes are triggered by rapidly changing stimuli in the sensory environment. Indeed, unnecessary facilitation of, for example, blinking has a cost: the probability of the individual to be harmed in other ways increases with the strength of blinking. Therefore, rapid enhancement or reduction of the facilitation of the blink reflex allows optimal avoidance of environmental threats.

### 4.2 HBR magnitude depends on the predicted stimulus location

Experiments 1 and 2 showed that the modulation of the HBR circuitry in the medulla occurs within tens of milliseconds. Experiment 2 yielded an important additional finding: the HBR is modulated according to a model that takes into account both (1) the actual position of the hand with respect to the face and (2) the *predicted* location of the hand. In Experiment 2 participants were required to *voluntarily* move the hand (and therefore the threat represented by the electrical stimulus) either towards or away from the face. The estimated position of the hand in external space during a voluntary movement is based on an internal forward model that reliably predicts the consequences of motor commands. Such a model relies on the motor command itself, as well as on the comparison between the predicted and the actual proprioceptive and visual feedback generated by the movement. Such continuous comparison allows precise estimation of limb position during self-paced movements (Wolpert & Flanagan, 2001). Therefore, participants were able to predict accurately the *direction* of hand movement, and the forthcoming hand position when the stimulus was delivered.

A related question is whether the proprioceptive and/or visual information alone (i.e., without the forward model generated by the voluntary movement) would result in similar predictions about hand locations, and, therefore, in similar HBR modulations. Performing the same paradigm of Experiment 2 while the hand is passively moved by an external source would allow addressing this point. A result similar to that reported here (Fig. 4) would indicate that sensory feedback alone is sufficient to make predictions about forthcoming hand position. Regarding the respective contribution of proprioceptive and visual feedback, previous experiments have shown that the ‘far–near effect’ is entirely unaffected when the eyes are closed or when the participants cannot see the hand (Sambo, Forster, et al., 2012). This suggests that proprioceptive information is sufficient to determine a HBR modulation similar to that observed with eyes open and during voluntary movement.

Stimuli delivered in the ‘Semi-far’ and ‘Semi-near’ positions while the hand was moved *away* from the face elicited HBR responses whose magnitude was larger when the stimulus was closer to the face (Fig. 1, Fig. 4). This ‘Semi-far’–‘Semi-near’ effect is reminiscent of the typical far–near modulation of the HBR magnitude, and its size was similar to that observed while delivering stimuli at similar distances from the face, but with the hand kept still for several seconds before receiving the stimulus (see the HBR elicited while the hand was in positions 2 and 3 in Sambo & Iannetti, 2013).

Crucially, when the hand was moved *towards* the face, the ‘Semi-far’–‘Semi-near’ effect vanished, because, in this movement direction, the magnitude of the HBR elicited by stimuli delivered while the hand was still away from the face (‘Semi-far’ position) was as large as that of the HBR elicited by stimuli delivered when the hand was closer to the face (‘Semi-near’ position, in both movement directions) (Fig. 4, lower panel). In contrast, when the hand was moved away from the face, there was a typical ‘Semi-far’–‘Semi-near’ difference (Fig. 4, middle panel). In other words, there was a clear dissociation between direction of the movement and HBR increase.

What could be the mechanism underlying such dissociation? A parsimonious explanation could be that the brain's ability to predict the position of limbs during voluntary movements is different as a function of the direction of movements: movements away from the body would result in inaccurate predictions. However, these predictions are not heavily dependent on movement direction (e.g., Wolpert, Diedrichsen, & Flanagan, 2011), and even possible differences in prediction accuracy would unlikely explain the dramatic difference observed in the two movement directions.

Alternatively, and more likely, there might be two interacting mechanisms: the evaluation of the actual hand position, and the prediction of its position during a voluntary movement. In other words, the models that the brain uses to decide the strength of the modulation of subcortical reflexes might be asymmetrically tuned: they yield a pre-emptive, stronger defensive response when there is a prediction that the threat will be closer to the body territory to be defended (i.e., in the present experiment, the HBR elicited when the hand is in the ‘Semi-far’ position and is moving towards the face, Fig. 1, Fig. 4, lower panel), but also when there is a prediction that the threat will move away from the face (i.e., in the HBR elicited when the hand is in the ‘Semi-near’ position and is moving away from the face, Fig. 1, Fig. 4, middle panel). This can be conceptualized as an additional “safety rule” in the model, that minimises the likelihood of responding with an HBR of normal (i.e., non-increased) magnitude when the threat is still close to the face.

Such asymmetric modulation is reminiscent of the observations of Zhao, Irwin, Bloedel, and Bracha (1999), who explored the conditioned anticipatory eye blink responses during hand movements towards or away from the face. They observed that only when the hand was quickly moved towards the face, a movement that eventually resulted in a tap of the forehead, an eye blink was generated before the forehead tap. Albeit the anticipatory eye blink described by Zhao et al. (1999) is an additional, independent eyelid response preceding the blink reflex induced by the actual trigeminal stimulation (and is therefore fundamentally different from the *facilitation* of the HBR that we measured in the present experiments), the direction-specificity of this phenomenon reflects the nervous system ability to make meaningful predictions about environmental threats and elicit appropriate defensive response. In this sense, their observation is similar to our finding that hand movements towards the face results in an upregulated HBR response even when the hand is still far away from the face (Fig. 1, Fig. 4).

A perhaps surprising observation is that the HBR elicited when the hand was in the ‘Semi-near’ position was similar in the two directions of movement (Fig. 4). The lack of a further increase of the ‘Semi-near’ HBR in the towards direction is probably due to a ceiling effect: when the threat content of the environmental situation is estimated to be high because of proximity with the defended area, the nervous system exert a maximal facilitation on the medullary circuitry subserving the blink response. Indeed, when the HBR is elicited in response to stimuli located in a number of spatial locations, an abrupt rather than a gradual increase of the HBR magnitude is observed with greater proximity of the hand to the face, and, accordingly, such distance-dependent modulation of HBR magnitude can be effectively modelled using a series of step functions (Sambo & Iannetti, 2013).

### 4.3 Conclusion

The present results indicate that the cortical modulation of the strength of the blink reflex occurs continuously, and takes into account the predictions about the spatial location of the stimulus in a purposeful manner: when the stimulus moves towards the body, and has therefore a higher threatening value, the blink reflex is anticipatorily upregulated. This real-time, predictive control of the excitability of subcortical reflex circuits ensures optimal behaviour in rapidly-changing sensory environments.

## Conflicts of interest

The authors declare no conflicts of interest.
